# News and Coming Events

**Published:** 1908-03-07

**Authors:** 


### NEWS AND COMING EVENTS.

Dr. F. L. Golla has been appointed assistant physician to St. George's Hospital.

Dr. M. A. Cassidy has been appointed resident assistant physician to St. Thomas's Hospital.

Mr. Gerald E. Friend has been appointed superintendent and resident medical officer at St. George's Hospital.

The council of King's College have elected Dr. J. Charlton Briscoe to the post of assistant physician to King's College Hospital.

Sir George Barham has accepted the invitation of the Tuberculosis (Animals) Committee to become a member of the Executive of that body.

Sir Walter Foster's Bill relating to public health officers was read for the first time in the House of Commons last week. The Bill is to amend the law relating to the qualification and tenure of office of medical officers of health and for other purposes.

Mrs. Frederick Gutteres Henriques has presented to St. Mary's Hospital a sum sufficient to provide for the complete equipment and furnishing of the new operating theatre in the Clarence Memorial Wing. This handsome gift will enable the board shortly to open this additional theatre, of which the hospital is in urgent need.

At the first meeting of the Royal Commission on Whisky, held on February 26 at the offices of the Local Government Board, it was decided that the first evidence should be that from the Excise, and that sittings should be held at the Westminster Palace Hotel on Monday, Tuesday, and Wednesday of this week. The proceedings of the Commission were open to the Press and to the public.

An inquest was opened at Slough on February 24, as to the death of Mrs. Margaret Edith Weston. Mrs. Weston had been medically attended for asthma and bronchitis, and, it was stated, had taken an inhalation containing cocaine. The inquiry was adjourned, pending the holding of a post-mortem examination. At the adjourned inquest held this week a verdict of death from natural causes was brought in.

The Earl of Northbrook, President of the Cancer Hospital, Fulham Road, occupied the chair, on February 26, at the fifty-seventh annual meeting of the Governors. The Committee stated that there had been a further increase in the amount of work done in the pathological and research department, and investigations were being carried on with a view of throwing light on the causation, spread, and prevention of disease. The number of cases of death after operations was 1.7 per hundred. Twelve beds have been set apart for the investigation of special treatments. Lord Northbrook said that an impression prevailed that remedies which might be effective in cancer cases were not tried because of the prejudice of medical men. That was not the case in that hospital; the medical staff was willing to receive any information which would help them to combat the disease and to try any remedy which had any prospect of success and did not endanger the patient. The result of the trial of most of these "remedies" had been very disappointing.

The annual meeting of the Chesterfield and North Derbyshire Hospital will be held in the Market Hall, Chesterfield, at 4.30 p.m., on Thursday, March 12. All subscribers are invited to attend.

An inquest was held at Brighton last week on the body of the Hon. Mrs. Elizabeth Meade, wife of Captain the Hon. E. B. Meade and youngest daughter of Lord Selby. It appeared from the evidence that Mrs. Meade had been in bad health for some time and had suffered from insomnia. To induce sleep she had been in the habit of taking drugs. While at Brighton she took cachous of trional, an overdose of which had caused her death. A verdict of "Death from misadventure" was returned.

The case of Dr. Hall-Edwards, of Birmingham, who has lost his left arm owing to his devotion to experimental work with the Röntgen rays, has been brought to the notice of the King, with the suggestion that his self-sacrifice is deserving of a Civil List pension. The following reply has been received from Lord Knollys: "I have had the honour of submitting your letter of February 26 and the enclosure which accompanied it to the King. I need hardly tell you that his Majesty warmly appreciates the self-sacrificing act of Dr. Hall-Edwards, but he thinks it would be more regular were you to communicate with the Prime Minister respecting the matter than to him."

Dr. Conolly Norman, medical superintendent of the Richmond Lunatic Asylum, Dublin, died suddenly in Dublin last week, in his fifty-fifth year. He was educated at Dublin University, and having acted as superintendent of several provincial asylums was appointed to the control of the Richmond Asylum, the premier lunatic asylum in Ireland. Dr. Norman instituted many reforms in the treatment of the patients in this institution and abolished, with the happiest results, the system of severe restraint and other relics of the old prison methods. He was successively secretary and president of the Medical Psychological Association, and joint editor of the *Journal of Mental Science*.

At Marlborough Street, on February 26, Isidore Zeifert, a Russian druggist, of Broad Street, Golden Square, was committed for trial on the charge of having caused the death of Mrs. Jane Farvish by administering cocaine to her. The facts of the case, briefly recalled, are that on January 18 Mrs. Farvish went to Zeifert's shop to have some teeth extracted, and he injected cocaine into the gum to deaden the pain. She fainted, and, in spite of all efforts to save her, died. Medical evidence as to the uses of cocaine was given by Dr. Dunn, police surgeon, and Dr. Freyberger, the latter stating that the post-mortem examination showed that the woman had a diseased heart. The accused pleaded "Not guilty," and reserved his defence for trial, Mr. Denman allowing bail. At the opening of the March Sessions at the Central Criminal Court on March 3 the Recorder, in his charge to the grand jury, referred to this case. His Lordship said, whether it was one that in the end would call for any punishment was not for him to say, but he thought, having regard to the fact that there were a great many unqualified persons practising as dentists for profit or gain, it was very desirable that they should be made to understand that, if they took on themselves duties which they were not fully competent to carry out, they would have to bear the consequences.

The King has sent an invalid couch to Guy's Hospital in recognition of the services rendered to one of his personal servants. The couch bears the following inscription: "Presented by the King to 'Cornelius' Ward, Guy's Hospital, 1907."

Miss Katharine Stewart Forbes, of Ascot, whose will has recently been proved, left £42,300 for charitable purposes, including £10,000 to the Brompton Hospital for Consumption, £10,000 to the Brompton Cancer Hospital, £5,000 to Middlesex Hospital for the cancer ward.

It is reported from Cardiff that what is believed to be anthrax has carried off a herd of fifteen cattle at Ynysmudw, and three men who had been engaged in the reburial of the carcasses at a greater depth and had opened one of them have been removed to hospital at Swansea seriously ill.

The eighteenth Congress of French Neurologists will be held at Dyon from August 3 to 9 next. The Congress will chiefly discuss "the diagnosis and clinical types of neuralgias, mental troubles due to defective function of glands possessing an internal secretion, and the treatment of mentally-defective children."

An anonymous donor has offered to Cambridge University £300 a year for five years towards the stipend of a new Professor of Biology, who should devote himself to those subjects which were the chief concerns of Charles Darwin's life work. The Council of the Senate recommend that the offer be gratefully accepted.

The annual conversazione of the West London Post-graduate College will be held on Wednesday, March 18, at the West London Hospital, Hammersmith Road, W. His Grace the Duke of Abercorn will hold a reception at 8.30 p.m. There will be music, exhibitions, etc., and refreshments will be provided. The Dean would be pleased to send a card of invitation to any medical man who would like to attend.

It is reported from Paris that the French Government has under consideration a decree more strictly regulating the sale of opium than has hitherto been the case. Henceforth, according to the terms of this decree, only such apothecaries and druggists as receive special authorisation may keep or sell opium. They must, further, ascertain the object for which the drug is purchased, and keep a register of that object, as well as of the name and address of the buyer and the quantity sold.

The next International Congress on Tuberculosis will take place at Washington, the meetings commencing on September 28 and ending on October 3 next. The Smithsonian Institute offers a prize of £300 for the best paper read or submitted on the influence of atmospheric air in the causation and spread of tuberculosis. Papers may be written in French, German, or English, and the award will be made by a special committee appointed by the Institute and the Congress.

The annual meeting of the London Homœopathic Hospital was held on February 28, the Right Hon. the Earl Cawdor, the Treasurer, presiding. The Board had to report with great satisfaction the completion of the Building Extension Fund of £30,000, which had all been paid and invested with the exception of £1,770 promised to be paid during 1908. Plans are now being prepared, and it is hoped soon to have the wards built for accommodating middle-class contributing patients.

On February 25 a stained-glass window was unveiled in the Dover Town Hall in memory of Dr. Astley, a well-known Kentish philanthropist.

Last week in London the death-rate, which had been 17.7, 19.0, and 18.5, fell to 16.8. The deaths directly attributable to influenza numbered 141, against, 34, 86, and 126 in the preceding three weeks. Out of these 141 deaths 86 occurred in persons of the age of 60 and upwards and 124 in persons over 40.

By virtue of special powers received from the Holy See, the Archbishop of Westminster, on account of the prevalence of influenza, has dispensed Roman Catholics of his diocese from the laws of fasting and abstinence during Lent, except on Ash Wednesday and Fridays.

Sir Philip Magnus on March 4 put the following question to Mr. Asquith (as representing the Prime Minister): "To ask the Prime Minister whether, having regard to the prevalence of influenza among members of the House of Commons, he will reconsider the advisableness of a daily adjournment of the House for a half-hour between the hours of seven and nine in the evening, with a view to the better ventilation of the House by the admission of fresh air through opened windows."

At a meeting of the Council of the University of Paris, held at the Sorbonne, the President presented a loving-cup given by the London University as a souvenir of the hospitality extended to it last summer. The cup, which was designed by Mr. Omar Ramsden and Mr. Alwyn Carr. is embellished with the arms of both Universities and of France and Great Britain, and with figures symbolising Science, Letters, and Art.

The Medical Officer of Health for the Port of London, in a report to the Corporation, draws attention to the recent seizure of a consignment of unsound "white of eggs," which was condemned as unfit for human food. On a visit being paid to the premises of a manufacturing confectioner, to whom two of the cases had been sold, it was found that the contents of the cases were actually being used in the manufacture of "nougat." The other four cases were destroyed. The medical officer for the Port states that this material seems to be much in request for the manufacture of cheap sweetstuff and confectionery for consumption among children.

The annual meeting of the Seamen's Hospital Society, which is responsible for the management of the *Dreadnought* at Greenwich, was held on February 28 under the presidency of Mr. Perceval A. Nairne. The Chairman deprecated the general tendency to condemn seamen as being reckless and thriftless. Their calling was harder than that of the labourer on shore, and it was the constant strain of working week in and week out which resulted in their often breaking down in health at a comparatively early age. More funds were needed to assist them in providing accommodation for sick and injured seamen who sought relief in the Port of London, the deficiency for the past year being £2,262. Lastly, Mr. Nairne spoke of the work of the two schools—the London School of Tropical Medicine and the London School of Clinical Medicine—carried on by the Society.

H.R.H. Princess Christian has accepted a copy of "The Way of the Wilderness," a threepenny booklet of readings for the sick, a third edition of which has been issued by Mr. J. S. Nicholas, 34 Brecknock Road, Bristol.

